# Apathy and Depression Impact on Cognitive Decline: Neuropsychological Specificities across Neurodegenerative Diseases

**DOI:** 10.1093/arclin/acaf121

**Published:** 2026-01-30

**Authors:** Serena Oliveri, Francesca Gastaldo, Natale Vincenzo Maiorana, Matteo Guidetti, Tommaso Bocci, Chiara Rosci, Sara Marceglia, Alberto Priori

**Affiliations:** Department of Health Sciences University of Milan, “Aldo Ravelli” Center for Neurotechnology and Brain Therapeutics, Via A. di Rudinì, 8—ASST Santi Paolo e Carlo—Corpo C, Milano, MI 20142, Italy; Neurological Clinic, ASST—Santi Paolo e Carlo Via A. di Rudinì, Milano, MI 20142, Italy; Neurological Clinic, ASST—Santi Paolo e Carlo Via A. di Rudinì, Milano, MI 20142, Italy; Department of Health Sciences University of Milan, “Aldo Ravelli” Center for Neurotechnology and Brain Therapeutics, Via A. di Rudinì, 8—ASST Santi Paolo e Carlo—Corpo C, Milano, MI 20142, Italy; Department of Health Sciences University of Milan, “Aldo Ravelli” Center for Neurotechnology and Brain Therapeutics, Via A. di Rudinì, 8—ASST Santi Paolo e Carlo—Corpo C, Milano, MI 20142, Italy; Department of Health Sciences University of Milan, “Aldo Ravelli” Center for Neurotechnology and Brain Therapeutics, Via A. di Rudinì, 8—ASST Santi Paolo e Carlo—Corpo C, Milano, MI 20142, Italy; Neurological Clinic, ASST—Santi Paolo e Carlo Via A. di Rudinì, Milano, MI 20142, Italy; Neurological Clinic, ASST—Santi Paolo e Carlo Via A. di Rudinì, Milano, MI 20142, Italy; Department of Health Sciences University of Milan, “Aldo Ravelli” Center for Neurotechnology and Brain Therapeutics, Via A. di Rudinì, 8—ASST Santi Paolo e Carlo—Corpo C, Milano, MI 20142, Italy; Department of Health Sciences University of Milan, “Aldo Ravelli” Center for Neurotechnology and Brain Therapeutics, Via A. di Rudinì, 8—ASST Santi Paolo e Carlo—Corpo C, Milano, MI 20142, Italy; Neurological Clinic, ASST—Santi Paolo e Carlo Via A. di Rudinì, Milano, MI 20142, Italy

**Keywords:** Apathy, Depression, Vascular dementia, Parkinson’s disease, Dopamine, Cognitive impairment

## Abstract

**Objective:**

Apathy is a common symptom across neurodegenerative diseases with origins still debated. “Vascular apathy hypothesis” by Van der Mast suggests vascular pathologies precede apathy. Other evidence points to dysfunction in dopamine pathways, driving apathy by impairing goal-directed behaviour. The impact of apathy on cognitive decline and autonomy, particularly with coexisting depression, remains unclear. This *cross-sectional study* aimed to (1) characterize apathy in vascular dementia, Parkinson’s disease (PD), and Mild Cognitive Impairment-Alzheimer Disease (MCI-AD) regarding incidence, severity, and cognitive specificity; (2) differentiate effects of apathy and depression on cognitive impairment and daily autonomy (Activities of Daily Living [ADL]).

**Method:**

Fifty-three patients underwent neuropsychological testing and completed the Geriatric Depression Scale, Starkstein’s Apathy Scale, and ADL questionnaire at a clinical neuropsychology outpatient setting in Milan.

**Results:**

56% patients had cardiovascular pathologies, 20% had PD, and 22% had MCI-AD. Neither prevalence nor severity of apathy or depression differed significantly across diseases**.** Hierarchical regression showed apathy predicted language initiative controlling for depression (R2 = 0.249; F(2) = 4.144; *p* = .028), and inversely correlated with working memory, language and frontal functioning, while depression predicted autonomy controlling for apathy (R2 = 0.234; F (2) = 3.821; *p* = .036).

**Conclusions:**

Apathy is prevalent across different neurodegenerative diseases and exacerbates specific cognitive impairments. Distinguishing vascular apathy from other forms remains challenging.

## INTRODUCTION

Apathy is a cross-cutting symptom in neurodegenerative diseases that often precedes the onset of cognitive and/or motor symptoms (Aalten et al., 2007; Ma, 2020; Zhao et al., 2016) and has been described in multiple disorders, such as Alzheimer’s disease, frontotemporal dementia, Huntington’s disease, major depressive disorder, Parkinson’s disease, schizophrenia, cerebrovascular disease, traumatic brain injury and vascular dementia (Fahed & Steffens, 2021). Apathy symptoms are also common in elderly general population (Caeiro et al., 2013; Groeneweg-Koolhoven et al., 2017; Lyketsos et al., 2002; Onyike et al., 2007; van Reekum et al., 2016).

Apathy is distinguished from depression by the specific presence of indifference, reduced motivation and initiative, and by the absence of other typical symptoms experienced by the depressed patient, such as sadness, hopelessness, guilt, and emotional suffering (Brodaty & Connors, 2020; Lanctôt et al., 2023a; Starkstein et al., 2005).

There are at least two main perspectives regarding the underlying aetiology of motivation disorders.

The first one, based on the “vascular apathy hypothesis” (van der Mast et al., 2008) postulates a close interplay between apathy and several proxies of cardiovascular disease (CVD): vascular pathologies have been supposed to precede apathy symptoms but not depressive ones (Lohner et al., 2017; Wouts et al., 2020) and apathy might also be a marker of subclinical (cerebral) small vessel disease (SVD) which then could result in overt CVD (Eurelings et al., 2019; van der Klei et al., 2022). Vascular pathologies potentially make older adults more susceptible to developing clinically significant apathy in later life, and to accelerating cognitive decline in vascular dementia (Mok et al., 2024). Various mechanisms could drive the vascular foundation of apathy, such as atherosclerosis or structural brain damage following stroke. A strong association has been found between symptoms of apathy and a history of stroke/transient ischemic attack (TIA), or cardiovascular risk factors among community-dwelling elderly participants who were free from depressive symptoms and dementia, suggesting an important contribution of vascular factors in the aetiology of apathy (Ligthart et al., 2012). Furthermore, Tay et al. (2020) already showed that apathy over time, unlike depression, is linked to dementia in patients with SVD, suggesting that apathy could serve as an early warning sign of dementia in SVD patients. Instead, Li et al. (2023) hypothesized that apathy is an independent contributor to cognitive impairment in SVD patient, and van Dalen et al. (2018a) demonstrated that apathy symptoms are associated with cognitive decline and dementia independently by history of cardiovascular disease or stroke.

On the other side, the recent model of apathy proposed by Marin, Starkstein, Levy, and Dubois (Levy & Dubois, 2006; Marin et al., 1991; Starkstein, 2005) recognizes different components of apathy, emotional, cognitive, and behavioural, which appear to converge on distributed dopaminergic circuits—particularly fronto-striatal, fronto-limbic and fronto-parietal pathways—whose dysfunctions may underlie the syndrome across different clinical populations. Emotional apathy (affective blunting, indifference, reduced affective involvement) would be mainly linked to sensitivity to reward stimuli, and mainly involves the mesolimbic dopaminergic pathway (Salamone & Correa, 2012), while cognitive/motor activation and energy investment (effort) required by a task/action are mainly modulated by the dorsolateral and nigro-striatal dopaminergic pathway (Le Bouc et al., 2016; Le Heron, Plant, et al., 2018).

In this framework, Parkinson's Disease provides fundamentally different substrates to investigate apathy, since it primarily arises from neurodegeneration affecting the dopaminergic system, the nucleus accumbens, the anterior cingulate cortex (ACC) and the dorsolateral prefrontal cortex, which are critical regions for motivation, reward processing, and goal-directed behaviour (Béreau et al., 2023) In Parkinson's disease (PD), apathy can be observed in the prodromal stage of the disease as well as in more advanced patients receiving bilateral chronic subthalamic nucleus stimulation (Zoon et al., 2023): in early stages apathy occurs as an a-motivational behavioural syndrome, while in advanced stages it may be considered as a cognitive apathy preceding cognitive decline and dementia (Béreau et al., 2023).

A meta-analysis conducted by D’Iorio et al. (2018) revealed a moderate relationship between apathy and global cognitive functioning, long-term verbal memory, processing speed/attention/working memory, visuospatial ability and selective executive functions (i.e., abstraction ability/concept formation, generativity and inhibition) in patients affected by PD.

Nonetheless, dopaminergic disfunction alone may be insufficient to explain apathy when it arises in conditions characterized by widespread network disconnection, such as in cerebrovascular disease or Alzheimer’s pathology (Joo et al., 2016) where apathy can occur before or alongside cognitive decline (Mehak et al., 2023), thus supporting the “vascular hypothesis”.

The distinct pathophysiological bases and patterns of symptoms progression in vascular dementia, PD and MCI-AD—linked respectively to cerebrovascular damage, dopaminergic and fronto-subcortical circuit dysfunction, or amyloid-tau deposits—suggest that apathy could manifest differently in each. However, comparative studies are limited or primarily focus on imaging comparisons (see the list of studies in the recent review by Steffens et al. (Steffens et al., 2022).

While it is clear the strong relationship between apathy and cognitive impairments, it is still to be determined whether the sequelae of cognitive impairment related to the vascular apathy hypothesis differs from that observable in patients characterized by dopaminergic dysfunctions, and whether an etiology-specific apathy profile can be identified. Such a specific characterization would allow to understand whether distinct cognitive structures of apathy may reflect shared or divergent mechanisms of cortical and subcortical dysfunction across neurodegenerative disorders. This approach is quite novel because it allows the identification of shared and unique clinical manifestations and potential neurobiological mechanisms underlying apathy, which could inform tailored interventions and improve diagnostic precision for such a complex neuropsychiatric symptom.

Moreover, the lack of consistent profiling of the relationship between apathy and cognitive impairments may be partly due to the type of cognitive assessments mostly used in the literature which were restricted to global screening scales administration, such as the Mini Mental State Examination or the cognitive function was measured with Montreal Cognitive Assessment (MoCA) that are overall indicative of global cognitive impairment, but do not have adequate domain-specific sensitivity (D’Iorio et al., 2018; Li et al., 2023). Conversely, extensive neuropsychological testing might help to define the pathway whereby apathy induces cognitive impairments both in SVD and vascular dementia in general and in other conditions, like PD, where the dopaminergic network is directly involved and apathy has been largely described.

In addition, discriminating between apathy as part of a depressive disorder, or apathy as an independent syndrome remains difficult in individuals coping with a neurodegenerative diseases, since anhedonia, loss of interest, indecisiveness and psychomotor retardation are common symptoms (Mouta et al., 2023b; Wouts et al., 2023).However, it has already been described that apathy is associated more specifically with deficits in executive functions such as planning, recall, and the ability to generate new cognitive strategies—problem solving, processes reliant on the frontal lobes and frontostriatal circuits; whereas depression may affect cognition but typically through different cognitive abilities, including attentional deficits and slowed processing, generating a typical condition known as “pseudodementia”(Lanctôt et al., 2023b; Mouta et al., 2023a; Varanese et al., 2011). Distinct neural circuits underlie apathy and depression in neurodegeneration. Apathy aligns with dysfunction in dopaminergic and frontal-subcortical circuits, whereas depression often involves limbic and serotonergic pathways. This difference translates into variable prognosis and treatment responses in cognitive decline trajectories (Dan et al., 2017; Steffens et al., 2022).

Extensive neuropsychological testing might also to define different cognitive sequelae between these two “overlapping” conditions.

For these reasons, in this *cross sectional study*, we aimed to (1) characterize apathy in different neurodegenerative diseases such as vascular dementia, Parkinson’s disease-PD and Mild Cognitive Impairment (MCI)-Alzheimer Disease (ad) in terms of incidence, severity, and specificity in cognitive decline, and (2) characterize the differential impact of apathy and depression on cognitive impairment and daily autonomy.

## METHODS

### Participants

Between November 2023 and June 2024, 53 patients were routinely evaluated (consecutive referrals) at the clinical neuropsychology and cognitive rehabilitation outpatient setting for diagnostic questions of cognitive impairment related to chronic vascular disease, Parkinson’s disease or MCI-ad (mild cognitive impairment, primarily in the memory domain suspected to be the preclinical stage of ad), at ASST Santi Paolo and Carlo Hospital in Milan, established based on diagnostics performed by neurologists belonging to the same unit. Patients provided informed consensus for the retrospective use of their anonymized clinical data for research purposes, ensuring respect for confidentiality and ethical standards. Ethical approval was obtained from the Milan Area 1 Ethics Committee to which ASST Santi Paolo e Carlo refers, with protocol number 0048450/2022 dated 21/11/2022.

Patients’ mean age was ~73 years, with an average of 9 years of education. Higher levels of education have been consistently shown to delay the progression of cognitive decline due to neurodegenerative diseases, generating a cognitive reserve that enhances the brain's resilience to pathology. Additionally, lower education levels have been associated with more severe cognitive impairment, apathy and depressive symptoms, reflecting neuropsychological vulnerabilities (Arora et al., 2024). None of the patients exhibited anosognosia, a neuropsychiatric condition characterized by a lack of awareness of cognitive deficits, neurological or psychiatric condition. However, this aspect was assessed qualitatively during the visits. All subjects underwent an extensive neuropsychological battery to assess cognitive domains. The Montreal Cognitive Assessment was used for global cognitive abilities evaluation (Santangelo et al., 2015); the Rey Auditory Verbal Learning Test (RAVLT) assessed long-term and short-term verbal memory for long-term and short-term verbal memory (Carlesimo et al., 2008); Digit Span Forward and Backward tested working memory (Orsini et al., 1987); the Rey–Osterrieth complex figure (ROCF) copy and recall assessed constructive apraxia abilities and long-term visuo-spatial memory (Caffarra et al., 2002a); the Visual Denomination Test (Sartori et al., 1993) evaluated naming abilities; Verbal fluency tests (Costa et al., 2014) assessed phonemic, semantic and alternate fluency including the shifting index; Visual Search measured selective attention (Spinnler & Tognoni, 1987), the Frontal Assessment Battery (FAB) evaluated executive functions (Appollonio et al., 2005); and the Stroop test (short version) assessed processing speed and inhibition of interfering stimuli (Caffarra et al., 2002b). Patients were also evaluated for daily living autonomy with the Activities of Daily Living (ADL) scale and for apathy and depression through specific scales, as detailed below. For each neuropsychological test, standardized correction tables were used to adjust scores for demographic variables such as age, education, and sex to improve the accuracy of test interpretation. These demographically corrected scores were then employed in all analyses to minimize confounding influences and ensure accurate interpretation of cognitive performance in the study population (delCacho-Tena et al., 2024).

### Assessment scales

#### Geriatric depression scale

The 30-item Geriatric Depression Scale (GDS) (Yesavage et al., 1982) is designed to measure depression in the elderly, primarily as a screening instrument. One well-known advantage of the GDS is its forced-choice (yes/no) response format, which requires very little cognitive involvement, making it especially helpful for patients with cognitive dysfunction. GDS total scores, calculated by counting responses that indicated depression, have a possible range from 0 (no depression) to 30 (severe depression). Clinical cutoffs are as follows: normal, 0–9; mild depression, 10–19; severe depression, 20–30.

#### Italian adaptation of the Starkstein’s Apathy Scale

The Starkstein’s Apathy Scale (SAS) is a 14-item rating scale specifically designed for the evaluation of cognitive, behavioral, and emotional symptoms of apathy in patients with PD (Starkstein et al., 1992). Since it originates from the Apathy Evaluation Scale (AES), which has been extensively applied for AD and MCI-AD (Starkstein et al., 2006) there is evidence supporting SAS applicability in MCI due to Alzheimer's Disease (MCI-AD) (Guercio et al., 2015). Applicability of SAS has been demonstrated post-stroke (Hum et al., 2021), but a direct validation specifically for vascular conditions is less clearly established (Bu et al., 2025).

Starkstein et al. identified an optimal cut-off score of ≥14, with patients scoring above this threshold classified as apathetic. They found the sensitivity to be 66% and the specificity 100% (Starkstein et al., 1992). In this study, we used the Italian adaptation of the SAS (Garofalo et al., 2021).

#### Activities of Daily Living

For this study, we used the ADL scale developed by Katz et al. (1963). The scale, designed to assess autonomy in the basic ADL, is one of the most widely used instruments in clinical settings. It accurately evaluates six basic activities: bathing, dressing, toileting, moving around, urinary and faecal continence, and feeding. The scores are dichotomous (dependent/independent), which makes the instrument less flexible than the Barthel's Index, especially in fragile subject populations such as patients with dementia. Despite these limitations, the widespread use of the Katz index allows for the assessment of autonomy levels in large populations and ensures consistent evaluations in longitudinal studies.

### Statistical analysis

We performed descriptive analyses to examine the distribution of participants according to gender, age, diagnosed pathology, level of depression, apathy and comorbidity of depression and apathy, given that apathy is included among the symptoms of depression in DSM-5. Contingency tables and Chi-square tests were used to assess the interaction between diagnosed neurological diseases and the distribution of apathy and depression. Additionally, non-parametric correlations were conducted using Spearman’s coefficient, and group comparisons were performed with the Wilcoxon-Mann–Whitney and Kruskal-Wallis tests to evaluate differences in neuropsychological task performance among groups (*p* < .05).

Finally, a hierarchical linear regression was performed to verify whether apathy scores could predict performances in specific neuropsychological tasks.

## RESULTS

### Descriptive statistics

The study sample was composed of 56% patients with cardiovascular pathologies (hypertension, high cholesterol, heart diseases), 20% patients with PD, and 22% with MCI-ad.

Among patients enrolled in this study 52% had no depression, 26% had mild depression, and 16% suffered from severe depression. We also found that 43% patients were non-apathetic, whereas 56% exhibited clinically significant apathy. Results for depression and apathy were combined to classify subjects (see Table 1) as non-apathetic and non-depressive patients (33%), patients with pure apathy (24%), patients with pure depression (9%), and patients with both depression and apathy (32%).

**Table 1:** Descriptive frequencies enrolled patients

	**M ± SD**
**Age**	73.736 ± 9.546
**Education**	9.546 ± 4.451
**Sex**	**N**
Female	31
Male	22
**Pathology**	
Vascular pathologies	30
Parkinson’s disease	11
MCI-AD	12
**Depression severity**	
Non-depressed	28
Mild	14
Severe	9
**Apathy level**	
Non-apathetic	23
Apathetic	30
**Apathy and depression**	
Non-apathetic, non-depressed	18
Pure apathy	13
Pure depression	5
Apathetic and depressed	17

No significant differences emerged in levels of apathy, depression, or cognitive test performance according to gender.

### Apathy and depression incidence in different pathologies

Contingency tables revealed no significant differences in the distribution of patients with pure apathy, pure depression, or both depression and apathy, depending on the diagnosed neurological disease (vascular, Parkinson, MCI) (χ^2^ = 4.438, df = 6, *p* = .618).

We also performed an ANOVA using Kruskal-Wallis Test, which showed no significant differences in apathy severity scores (SAS scale) (H = 0.827, *p* = .661) or depression scores (H = 0.995, *p* = .608) across the different pathologies.

### Cognitive impairment profile in different pathologies

Results of one-way ANOVA (factor: pathology), conducted using the Kruskal-Wallis test, showed similar performances across most tests and cognitive domains according to the neurological disease. An exception was found in the Rey Auditory Verbal Learning Test—long-memory (H = 8.473, *p* = .014), where patients with vascular dementia had significantly lower scores (Mean_V = 4.9; SD = ±3.2) compared to patients with PD (Mean_P = 7.7; SD = ±2.9) or MCI-ad (Mean_M = 7.8; SD = ±3.5) (see Table 2).

**Table 2:** Differences in cognitive impairment due to pathology

**Neuropsychological test**	**Group mean (SD)**			**Kruskal-Wallis test**	**p-value**
	**Vascular pathologies**	**Parkinson’s disease**	**MCI-ad**		
**MoCA**	20.0 (±5.9); N = 30	22.7 (±2.9); N = 11	24.0 (±2.3); N = 11	5.9	0.053
**RAVLT short term memory**	33.1 (±7.2); N = 27	36.9 (±7.8); N = 11	37.1 (±10.2); N = 12	2.5	0.289
**RAVLT long term memory**	4.9 (±3.2); N = 28	7.7 (±2.9); N = 11	7.8 (±3.5); N = 12	8.5	0.014^*^
**Span forward**	5.3 (±0.8); N = 30	5.0 (±1); N = 11	5.8 (±1.3); N = 12	1.7	0.417
**Span backward**	4.1 (±1.4); N = 30	4.2 (±1.1); N = 11	4.7 (±1); N = 12	3.1	0.216
**Rey figure**—**copy**	30.9 (±9.1); N = 27	27.5 (±7.7); N = 11	29.1 (±7.9); N = 12	4.3	0.114
**Rey figure—recall**	12.2 (±5.9); N = 25	13.0 (±4.9); N = 11	13.6 (±7.5); N = 12	0.5	0.774
**Phonemic fluency**	29.8 (±14.7); N = 29	25.3 (±4.8); N = 11	29.9 (±12.9); N = 12	1.7	0.423
**Semantic fluency**	35.6 (±8.0); N = 29	37.0 (±8.8); N = 11	38.9 (±8.2); N = 12	1.6	0.457
**Visual denomination test**	57.9 (±9.7); N = 27	61.8 (±3.1); N = 11	60.7 (±3.9); N = 12	2.7	0.252
**Frontal Assessment Battery**	14.5 (±3.3); N = 30	15.9 (±2.7); N = 11	17.2 (±3.5); N = 12	3.7	0.154
**Visual search for selective attention**	40.4 (±11.4); N = 30	42.6 (±7.0); N = 11	46.9 (±8.1); N = 12	2.7	0.256
**Stroop errors**	3.2 (±6.8); N = 27	1.0 (±1.7); N = 11	0.7 (±1.8); N = 12	0.2	0.883
**Stroop time**	48.0 (±46.7); N = 27	32.3 (±12.2); N = 11	28.4 (±18.4); N = 12	2.9	0.233
**Alternate fluency**	24.9 (±7.5); N = 26	23.9 (±7.1); N = 11	23.3 (±11.7); N = 11	0.4	0.805
**Shifting index**	0.7 (±0.2); N = 26	0.5 (±1.1); N = 11	0.7 (±0.3); N = 11	0.8	0.656

^*^p < .05; ^**^ p < .01

Also, performance on cognitive tests revealed no significant differences due to gender.

### Impact of apathy and depression on cognitive impairment across pathologies

We observed specific correlations (Spearman's rank correlation coefficient) between SAS scores and neuropsychological performances. SAS scores were inversely correlated with backward digit span (ρ = −0.299, *p* = .030, SE = 0.143, IC 95% [−0.527, −0.031]), phonemic fluency (ρ = −0.289, *p* = .038, SE = 0.145, IC 95% [−0.521, −0.018]), semantic fluency (ρ = −0.412, *p* = .002, SE = 0.146, IC 95% [−0.616, −0.157]), denomination (ρ = −0.364, *p* = .009, SE = 0.148, IC 95% [−0.583, −0.096]) and FAB (ρ = −0.372, *p* = .006, SE = 0.144, IC 95% [−0.583, −0.113]). These results indicate that higher levels of apathy are associated with poorer performance in working memory, verbal fluency, naming abilities, and frontal executive functioning (dysexecutive syndrome).

Similarly, investigations of correlations between GDS scores and neuropsychological tests showed that GDS scores were inversely correlated with semantic fluency (ρ = −0.307, *p* = .034 SE = 0.151, IC 95% [−0.544, −0.025]), ROCF copy (ρ = 0.368, *p* = .012, SE = 0.155, IC 95% [−0.595, −0.087]) and ADL scores (ρ = −0.315, *p* = .029, SE = 0.151, IC 95% [−0.550, −0.034]). These findings suggest that higher depression levels negatively affect semantic fluency, constructive apraxia, and daily autonomy.

Group comparisons using the Kruskal-Wallis’s test revealed no significant differences in neuropsychological test scores among patients with pure apathy, pure depression, or combined depression and apathy (see Table 3).

**Table 3:** Differences in cognitive impairment due to apathy and depression

**Neuropsychological test**	**Group mean (SD); N**				**Kruskal-Wallis test**	**p-value**
	**Pure apathy**	**Pure depression**	**Depression plus apathy**	**Non apathetic or depressed**		
**MoCA**	20.2 (±5.2); N = 13	21.0 (±2.7); N = 5	21.1 (±5.3); N = 17	22.8 (±5.1); N = 17	4.9	0.175
**RAVLT short term memory**	32.0 (±7.09); N = 12	37.1 (±6.8); N = 5	34.4 (±9.1); N = 15	36.6 (±8.4); N = 18	2.7	0.437
**RAVLT long term memory**	4.5 (±3.4); N = 12	8.6 (±2.6); N = 5	6.9 (±3.5); N = 16	6.0 (±3.5); N = 18	5.6	0.133
**Span forward**	5.3 (±0.8); N = 13	5.7 (±2.3); N = 5	5.2 (±0.8); N = 17	5.5 (±0.8); N = 18	1.1	0.785
**Span backward**	3.9 (±0.9); N = 13	4.2 (±0.8); N = 5	4.1 (±1.4); N = 17	4.7 (±1.4); N = 18	3.0	0.390
**Rey figure—copy**	30.0 (±10.2); N = 13	31.0 (±8.5); N = 5	28.0 (±8.4); N = 14	30.8 (±7.8); N = 18	2.3	0.514
**Rey figure—recall**	11.6 (±7.1); N = 11	16.9 (±2.2); N = 5	12.9 (±5.3); N = 14	12.1 (±6.4); N = 18	4.3	0.235
**Phonemic fluency**	28.8 (±8.5); N = 13	26.1 (±4.9); N = 5	24.0 (±10.6); N = 16	34.0 (±16.7); N = 18	3.2	0.358
**Semantic fluency**	35.3 (±6.2); N = 13	37.1 (±6.1); N = 5	33.5 (±7.7); N = 16	40.3 (±9.3); N = 18	6.0	0.111
**Visual denomination test**	59.8 (±5.4); N = 13	60.2 (±4.2); N = 5	57.3 (±11.5); N = 17	61.2 (±3.9); N = 15	2.4	0.485
**Frontal Assessment Battery**	14.4 (±3.4); N = 13	16.5 (±0.9); N = 5	15.0 (±4.3); N = 17	16.2 (±2.7); N = 18	6.1	0.106
**Visual search for selective attention**	44.0 (±8.1); N = 13	45.8 (±7.2); N = 5	39.5 (±11.3); N = 17	42.8 (±11.1); N = 18	1.5	0.676
**Stroop errors**	5.2 (±8.5); N = 11	0.8 (±1.1); N = 5	1.2 (±2.0); N = 16	1.4 (±4.9); N = 18	3.3	0.348
**Stroop time**	38.0 (±30.3); N = 11	34.1 (±20.8); N = 5	40.1 (±19.3); N = 16	42.3 (±53.7); N = 18	2.7	0.441
**Alternate fluency**	23.9 (±6.2); N = 12	25.2 (±9.2); N = 5	22.8 (±8.3); N = 14	25.6 (±10.0); N = 17	0.6	0.895
**Shifting index**	0.7 (±0.2); N = 12	0.7 (±0.2); N = 5	0.8 (±0.3); N = 14	0.5 (±0.8); N = 17	1.9	0.593

^*^p < .05; ^**^ p < .01

**Fig. 1:**
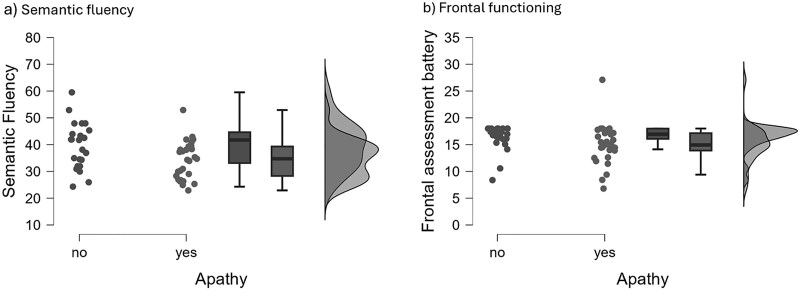
Semantic fluency and frontal functioning scores in apathetic vs non apathetic patients.

Nevertheless, by comparing patients with full blown apathy symptoms and patients with non-apathetic symptoms (by using SAS cut off 14 for distinguishing the two groups, Wilcoxon Mann Whitney test, U) the first group showed a significant worse performance in semantic fluency (Mean non apathetic = 39.596, SD = ±15.223; Mean apathetic = 34.289, SD = ±6.996) (Fig.  1a) and FAB (Mean non apathetic = 16.291, SD = ±2.423; Mean apathetic = 14.769, SD = ±3.882) (Fig.  1b). No significant differences emerged for all the other neuropsychological tests (Table 4).

**Table 4:** Differences in cognitive impairment between apathetic vs non apathetic patients (SAS scale cut off)

**Neuropsychological test**	**Group mean SAS score (SD); N**		**Mann Whitney test**	**p-value**
	**Non apathetic**	**Apathetic**		
**MoCA**	22.4 (±4.7); N = 22	20.7 (±5.2); N = 30	404.5	0.168
**RAVLT short term memory**	36.7 (±7.8); N = 23	33.3 (±8.1); N = 28	388.0	0.135
**RAVLT long term memory**	6.6 (±3.4); N = 23	5.9 (±3.6); N = 28	357.5	0.507
**Span forward**	5.5 (±1.2); N = 23	5.2 (±0.8); N = 30	395.5	0.369
**Span backward**	4.6 (±1.3); N = 23	4.0 (±1.2); N = 30	431.0	0.105
**Rey figure—copy**	30.8 (±7.7); N = 23	28.8 (±9.1); N = 27	354.0	0.397
**Rey figure—recall**	13.1 (±6.1); N = 23	12.3 (±6.1); N = 25	336.0	0.322
**Phonemic fluency**	32.3 (±15.2); N = 23	26.2 (±9.9); N = 29	390.5	0.298
**Semantic fluency**	40.0 (±8.7); N = 23	34.2 (±7.0); N = 29	456.5	0.024^*^
**Visual denomination test**	61.0 (±3.9); N = 20	58.4 (±9.3); N = 30	362.0	0.218
**Frontal Assessment Battery**	16.3 (±2.4); N = 23	14.8 (±3.9); N = 30	480.5	0.015^*^
**Visual search for selective attention**	43.5 (±10.3); N = 23	41.5 (±10.1); N = 30	395.5	0.370
**Stroop errors**	1.3 (±4.3), N = 23	2.8 (±5.8); N = 27	246.5	0.136
**Stroop time**	40.5 (±48.1); N = 23	39.2 (±23.9); N = 27	238.5	0.164
**Alternate fluency**	25.5 (±9.6); N = 22	23.3 (±7.3); N = 26	314.5	0.562
**Shifting index**	0.5 (±0.7); N = 22	0.7 (±0.2); N = 26	240.0	0.346

^*^p < .05; ^**^ p < .01

Data showed that patients with mild depression had greater difficulties in the forward span task compared to non-depressed patients or those with severe depression (H = 7.058; *p* < .029), although this finding was not confirmed by Dunn's post hoc test. Results of groups comparisons for each task are described in Table 5.

**Table 5:** Differences in cognitive impairment among depression severity levels (GDS ranges)

**Neuropsychological test**	**Group mean GDS score (SD); N**			**Kruskal-Wallis test**	**p-value**
	**No Depression**	**Mild Depression**	**Severe Depression**		
**MoCA**	22.1 (±4.6); N = 27	22.8 (±2.8); N = 14	18.8 (±6.3); N = 9	2.5	0.287
**RAVLT short term memory**	34.7 (±7.8); N = 28	35.8 (±7.7); N = 14	35.3 (±10.9); N = 7	0.3	0.858
**RAVLT long term memory**	5.3 (±3.5); N = 28	7.5 (±3.4); N = 14	7.3 (±3.2); N = 8	4.4	0.112
**Span forward**	5.5 (±0.7); N = 28	5.0 (±1.5); N = 14	5.6 (±0.8); N = 9	7.1	0.029^*^
**Span backward**	4.5 (±1.3); N = 28	4.4 (±0.9); N = 14	3.7 (±1.6); N = 9	3.9	0.144
**Rey figure—copy**	31.3 (±7.8); N = 28	28.4 (±9.3); N = 14	29.6 (±4.7); N = 6	2.2	0.333
**Rey figure—recall**	12.3 (±6.7); N = 27	14.7 (±5.7); N = 14	12.1 (3.0); N = 6	0.8	0.656
**Phonemic fluency**	33.3 (±13.8); N = 28	24.1 (±9.6); N = 14	24.6 (±3.3); N = 8	5.5	0.065
**Semantic fluency**	39.2 (±8.2); N = 28	35.0 (±7.5); N = 14	33.2 (±2.4); N = 8	4.8	0.089
**Visual denomination test**	60.9 (±4.0); N = 26	60.8 (±4.1); N = 13	53.9 (±14.8); N = 9	4.3	0.116
**Frontal Assessment Battery**	15.7 (±2.8); N = 28	15.8 (±1.6); N = 14	14.8 (±5.8); N = 9	2.1	0.359
**Visual search for selective attention**	43.9 (±10.0); N = 28	42.6 (±8.3); N = 14	38.0 (±13.2); N = 9	1.5	0.468
**Stroop errors**	3.1 (±6.8); N = 27	0.9 (±1.8); N = 14	1.2 (±1.8); N = 8	0.8	0.682
**Stroop time**	41.4 (±47.2); N = 27	38.6 (±19.0); N = 14	37.294 (±20.4); N = 8	1.7	0.417
**Alternate fluency**	25.1 (±8.7); N = 27	24.1 (±6.8); N = 14	22.6 (±11.6); N = 6	0.3	0.875
**Shifting index**	0.6 (±0.7); N = 27	0.8 (±0.2); N = 14	0.7 (±0.3); N = 6	0.9	0.632
**ADL**	5.8 (±0.4); N = 27	5.6 (±0.5); N = 14	4.9 (±1.8); N = 9	5.5	0.064

^*^p < .05; ^**^ p < .01

Finally, we conducted a hierarchical linear regression to examine whether apathy scores could predict phonemic fluency, semantic fluency, span backward, denomination and FAB scores, tasks with which levels of apathy were inversely correlated. We selected only patients classified as pure apathetic and apathetic and depressed individuals, for a total of 30 subjects. Apathy symptoms, as assessed by the SAS, were entered into the first step of the model and depression scores were entered in the second step, with neuropsychological scores as the dependent variable. We found no significant results regarding phonemic and semantic fluency, span backward and FAB scores. Instead for denomination (visual denomination test), results indicated that apathy is a significant predictor of denomination PC performance, while depression does not explain additional variance once apathy is considered.

In model 0 apathy significantly predicted denomination, (B = −0.95, t(26) = −2.86, 95% CI [−1.63, −0.27], accounting for 23.9% of the variance (R^2^ = 0.239, F(1,26) = 8.16, *p* = .008, (B = −0.95, t(26) = −2.86, 95% CI [−1.63, −0.27]) corresponding to a large effect size (f^2^ = 0.33; Cohen's *d*). In model 2 depression was entered after apathy. The addition of depression did not significantly improve the model, (Δ*R^2^* = 0.010, *p* = .566). In this final model, apathy remained a significant negative predictor, *B* = −0.84, *p* = .035, 95% CI [−1.63, −0.07], whereas depression was not a significant predictor, *B* = −0.14, *p* = .566, 95% CI [−0.61, 0.34].

Collinearity diagnostics indicated no issues with multicollinearity, with Tolerance values of 0.79 and VIF values of 1.27 for both predictors.

On the same subjects, we conducted a second hierarchical linear regression to examine whether depressive symptoms could predict neuropsychological tests scores, after controlling for apathy scores. We found no significant results for the cognitive domains. The overall model, instead, significantly predicted the autonomy in daily activities after controlling for apathetic symptoms.

In Model 0, depression significantly predicted ADL (*B* = −0.06, *t*(26) = −2.55, *p* = .017, 95% CI [−0.11, −0.01], explaining 20.0% of the variance (*R^2^* = 0.200, *f^2^* = 0.25).

In Model 1, apathy was added to the regression. The overall model accounted for 23.4% of the variance (*R^2^* = 0.234), but the increase in explained variance was not significant (Δ*R^2^* = 0.034, *p* = .300, *f^2^* = 0.044, small effect). In this model, apathy was not a significant predictor (*B* = 0.05, *p* = .300, 95% CI [−0.05, 0.14]). Again, collinearity diagnostics indicated no issues with multicollinearity, with Tolerance values of 0.79 and VIF values of 1.27 for both predictors.

## DISCUSSION

With apathy increasingly recognized as a transdiagnostic neuropsychiatric syndrome across many neurological and psychiatric conditions (Kos et al., 2016) and as an additional risk factor for cognitive decline (van Dalen, van Wanrooij, et al., 2018b), our study aimed to verify the existence of specific cognitive impairment profiles associated with the cerebrovascular origin of apathy, compared to other diseases where apathy symptoms are common (Parkinson’s disease and MCI due to probable AD), also considering the co-existence of depressive symptoms. Our results did not show any significant difference in the incidence or severity of apathy attributable to specific clinical pathologies, even when considering the subdivision between purely apathetic and apathetic-depressed subjects, nor were there differences in depression incidence across disease groups.

Nevertheless, our findings showed that, independent of neurological condition, high levels of apathy corresponded with poorer performance on tasks related to frontal functioning. Specifically working memory, language production (fluency and anomia, word retrieval), mental flexibility, motor programming, resistance to interference, and inhibitory control (measured by the FAB) were adversely affected.

The “vascular apathy hypothesis” (van der Mast et al., 2008) historically emphasized the role of cerebrovascular pathology—especially subcortical ischemic lesions—in the emergence of apathetic symptoms, while recent studies suggest that apathy more broadly reflect dysfunction of frontal-subcortical circuits (Le Heron et al., 2018a). Apathy was generally found in disorders involving the cortex in 60% of cases, compared to 40% of cases with subcortical disorders (van Reekum, 2005). Wouts et al. (2023) already addressed the limitations of the vascular apathy hypothesis and the challenges of identifying a specific apathetic profile in vascular patients.

Our findings support the hypothesis that apathetic states result from impaired engagement of executive control and action-initiation circuits, rooted in frontal and subcortical connectivity (Hachinski et al., 2022).

The inverse correlation of apathy with working memory, executive functions, and cognitive control mechanisms such as response inhibition and mental flexibility is consistent with the role these cognitive functions play in motivated behaviours and their underlying neural circuits (Le Heron et al., 2018b; Miyake et al., 2000; Stella et al., 2014). Lower performance on the FAB scale in apathetic patients has also been reported by Rea et al. (Rea et al., 2015), in a study comparing patients with Alzheimer’s disease patients with apathy during a drug trial combining donepezil and a cholinergic precursor. Similarly, Aiello et al. (Aiello et al., 2023) observed these findings in Parkinson’s disease patients with “pure” apathetic features, and Hachinski et al. (Hachinski et al., 2022) reported them in elderly individuals suffering from hypertension.

However, in our study, we also obtained clinical evidence showing how apathy can impair cognitive tasks that require language initiation and performance, such as fluency tasks and denomination (Cohen et al., 1999). Word production anomia, commonly known as the “tip of the tongue” phenomenon, can occur in everyday conversations or in patients with frontal lesions or Broca's aphasia, resulting in an inability to spontaneously produce a word or respond to a stimulus. These patients are usually aware of their naming difficulty and often report knowing the word. In many cases, prompts or cues can aid in word retrieval. It has been proposed that word production is akin to initiating nonverbal movements, a process that may be impaired in individuals with frontal lobe lesions (Kertesz, 2003).

Therefore, our neuropsychological findings support the hypothesis that apathy is underpinned by impairments in specific areas of frontal-subcortical circuitry, even in clinically mixed populations (vascular, Parkinson’s or AD). This impairment is particularly related to damage in the dorsomedial prefrontal cortex (DMPFC) circuit, originating in the ACC—a prominent hub of both cognitive and reward/emotional processing (Bonelli & Cummings, 2007).

Depression, which is often misdiagnosed as apathy, likely involves different brain structures such as the cingulate cortex, thalamus (Starkstein et al., 2009; Zahodne et al., 2013) and reduced serotonin (5-HT) transmission in the posterior cingulate and amygdala-hippocampus complex (Benoit & Robert, 2011). In our sample, was found to affect functions associated with short-term memory (digit span) and overall daily autonomy.

We found that depression also correlated with worse performance in semantic verbal fluency in our sample. The reason behind this correlation, in our opinion, differ from those described for apathy. Indeed, as shown by studies and meta-analyses (Fishman et al., 2019; Henry & Crawford, 2004), semantic fluency tasks recruit more extensive networks involved in information retrieval from semantic memory (temporal lobe), where information is stored as concepts, general facts, and vocabulary. Thus, in patients with depression, semantic fluency tasks may be indirectly affected by non-frontal impairments in long-term memory or word knowledge.

In our study, the involvement of memory in depression is also demonstrated by the scores obtained by mildly depressed patients in the digit span forward (short verbal memory), which were more deficient than those of patients without depression. It is also well-known that depression is a leading cause of memory loss in the elderly population (Mars & Marwaha, 2025).

In our study, depression was predictive of autonomy in everyday life, as suggested by a strong inverse correlation between GDS and ADL. The lack of autonomy in everyday activities was more significant with higher levels of depression. Indeed, studies have found that disability in household activities is associated with depressive symptoms, as it often leads to loss of independence (Feng et al., 2021). ADL disability and depressive symptoms appear to be mutually reinforcing over time (Ormel et al., 2002).

The relationship between depression and ADL in cardiovascular diseases can be explained as multifactorial. Depression may impact risk factors in cardiovascular patients, such as diet, low levels of physical activities, substance use, a higher smoking rate, as well as adherence to medical prescriptions (Krittanawong et al., 2023; Lang et al., 2022; Qi et al., 2024; Schuch et al., 2017). A meta-analysis conducted by Krittanawong et al. (2023) showed a significant increase in the risk of myocardial infarction and all cardiovascular diseases, as well as cardiovascular mortality, with an increased risk of incident stroke and heart failure. The increased likelihood of major vascular events could lead to disabilities, resulting in lower ADL scores and more severe depressive symptoms.

There are some limitations in this study. First, our sample size was small (53 patients), and the various pathologies were not equally distributed. It should be noted that the analyses, although statistically robust and promising, are to be considered exploratory due to the limited number of subjects per group. In the future, a larger and more homogeneous sample is needed to investigate further whether and how different pathologies might influence distinct manifestations of apathy correlated with specific neuropsychological outcomes. Additionally, the mean age of our sample was 73 years, which can be associated with a decline in physical function and difficulties in performing daily activities independently, potentially exacerbating the observed symptoms (Yan et al., 2023).

In conclusion, our results support the hypothesis that apathy is a transversal symptom across diverse cohorts of patients with neurodegenerative conditions, and unequivocally distinguishing vascular apathy and its cognitive sequelae from apathy in other clinical conditions may be challenging. Apathy appears to be a clinical condition that exacerbates impairments in specific cognitive functions more than depression, which partially affects short-term memory and daily autonomy. We also provide preliminary evidence suggesting that apathy might explain anomies, while depression might be a factor affecting patients’ dependency on caregivers for personal care. Therefore, screening for apathy and depression in neurological patients is crucial, as they present distinct neuropsychological profiles.

## Data Availability

The data supporting the findings of this study are available from the corresponding author upon reasonable request. Data sharing will be considered in accordance with applicable ethical guidelines, privacy regulations, and any restrictions related to participant confidentiality. Requesters may be asked to sign a data sharing agreement to ensure appropriate use and protection of the data.
